# The effect of dynamic stimuli on attention under different perceptual loads

**DOI:** 10.1186/s40101-025-00398-3

**Published:** 2025-07-04

**Authors:** Yuanli Li, Yoshihiro Shimomura

**Affiliations:** 1 Graduate School of Science and Engineering, Chiba University, 1-33 Yayoi-Cho, Inage-Ku, Chiba City, 2638522 Japan; 2 Design Research Institute, Chiba University, 1-33 Yayoi-Cho, Inage-Ku, Chiba City, 2638522 Japan

**Keywords:** Perceptual load, Dynamic stimulus, Event-related potential, Attention

## Abstract

**Background:**

Perceptual load is a major determinant of visual attentional selection patterns, and dynamic stimuli are salient bottom-up distractors. The present study investigated how dynamic stimuli, presented under different perceptual loads, impact the process of visual attentional selection.

**Methods:**

Fourteen participants (8 females and 6 males) were measured on task performance (reaction time and correctness) and event-related potentials while searching for visual exploratory task in a perceptual load paradigm.

**Results:**

In terms of behavioral performance, longer reaction times were required for the visual exploratory task under high perceptual load, whereas a trend suggestive of attentional capture reversal emerged under low perceptual load. Regarding ERP components, the P1 amplitude was more positive in the response to dynamic stimuli, while the N1 amplitude was more negative when dynamic stimuli were absent. The P3 amplitude was more positive in the presence of dynamic stimuli than in their absence and was also more positive under low perceptual load than under high perceptual load.

**Conclusions:**

This study found that N1 and P1 components were more sensitive to dynamic stimuli and insensitive to perceptual loads, while the P3 component effectively assessed both perceptual loads and dynamic stimuli. These variations reflect differential attentional allocation. Based on these findings, adapting interface displays according to gaze direction and perceptual load level can inform the design of user interfaces, such as those in navigation systems, educational materials, and assistive devices.

**Trial registration:**

This study was approved by the Ethics Committee of Chiba University Graduate School of Engineering (acceptance number: R4-20, Acceptance date: March 22, 2023).

## Introduction

Dynamic visual cues, due to their highly efficient attention-capturing properties, are widely applied in safety–critical domains such as in-vehicle systems and medical monitoring. For example, automotive head-up displays use flashing warning symbols to alert drivers to potential hazards, while intensive care units employ dynamic visual alarms to highlight sudden changes in patient conditions. These designs rely on the bottom-up attention capture mechanism induced by dynamic stimuli to enhance information processing efficiency [1]. However, in high cognitive load tasks—such as driving in complex traffic conditions or monitoring multiple medical parameters—dynamic stimuli may excessively consume limited attentional resources, leading to delayed reactions and decision-making errors. This phenomenon raises the possibility that the effectiveness of dynamic stimuli may depend on the perceptual load level of the current task, although the underlying neural regulatory mechanisms remain unclear and warrant further investigation.

Human survival in complex environments necessitates dynamic attentional resource allocation to accommodate rapidly changing informational demands while preserving cognitively efficient and ergonomically comfortable states. This adaptive process is governed by neurophysiological mechanisms that continuously negotiate a trade-off between selective information sampling and distractor suppression, particularly under fluctuating perceptual load conditions. By elucidating how dynamic stimuli modulate this equilibrium, our study aims to uncover the computational principles underlying the visual system’s environmental adaptability.

The attentional control model posits that visual selection emerges from the dynamic interaction between goal-directed (top-down) and stimulus-driven (bottom-up) mechanisms [2]. Perceptual load theory further suggests that task complexity modulates this balance by regulating the availability of attentional resources: high-load tasks fully engage resources, enforcing early selection to suppress distractions, whereas in low-load conditions, excess resources"spill over"to irrelevant stimulus processing, amplifying distraction [3]. While this framework has been extensively validated with static stimuli, its applicability to dynamic stimuli remains debated.

Critically, the disruptive effects of dynamic stimuli may be spatially modulated by visual processing characteristics. Although peripheral vision has lower spatial resolution than central vision, it exhibits significantly heightened sensitivity to motion and flicker [4]. This trait makes dynamic distractors particularly intrusive in the periphery, a phenomenon that likely amplifies attentional capture and task interference in real-world settings. The current study directly builds on this by assessing whether such peripheral sensitivity differentially impacts visual performance under dynamic conditions, extending prior findings from central-vision–dominated paradigms to more ecologically valid peripheral scenarios.

Research has demonstrated that perceptual load modulates attentional allocation through distinct event-related potential (ERP) components. The P1 component (70–130 ms latency), generated in the occipital cortex, reflects early sensory gating mechanisms. For instance, Lavie and colleagues found that high perceptual load significantly suppresses P1 amplitudes to peripheral distractors, indicating enhanced filtering of primary visual processing under resource competition [5, 6]. The N1 component (100–150 ms latency), localized to the temporoparietal junction, is closely associated with spatial attention allocation and stimulus discrimination. Under low load, task-irrelevant stimuli still elicit pronounced N1 negativity, suggesting automatic “spillover” of residual resources to distractor localization [7]. The P3 component (300–500 ms latency), originating from the parietal-anterior cingulate network, shows reduced amplitude under high load due to limited cognitive resources for distractor evaluation [8], while prolonged latency quantifies decision-making delays induced by perceptual load [9]. These findings highlight the unique utility of ERPs in dissecting hierarchical load effects: P1/N1 indexes early sensory-attentional modulation, whereas P3 tracks late-stage cognitive resource competition, collectively mapping a continuum from sensory filtering to cognitive suppression. However, existing ERP studies have predominantly utilized static, centrally presented stimuli, limiting our understanding of how dynamic distractors in the visual periphery modulate neural responses, particularly early sensory and late cognitive components.

This study examines the effect of dynamic stimuli on attention under different perceptual loads by integrating Lavie’s perceptual load paradigm [10] with event-related potential (ERP) techniques. Through manipulating visual search task complexity (low vs. high perceptual load) and measuring behavioral performance (reaction time and accuracy) along with P1/N1/P3 components, the study investigates the relationship between perceptual load and behavioral interference effects of dynamic stimuli, the role of early sensory components (P1/N1) in gating dynamic feature processing, and the involvement of higher-order cognitive components (P3) in resource-driven suppression.

## Method

### Participants

Sixteen adults with normal or corrected-to-normal vision participated in the experiment; of the 16, 2 with excessive noise in the EEG measurements were excluded from the analysis, leaving 14 participants for the analysis (8 females and 6 males; mean age 25.3 years, standard deviation 1.9 years). The study participants avoided strenuous exercise and alcohol on the day before the experiment and abstained from caffeine and smoking on the day of the experiment.

The participants were informed of the purpose and procedures of the experiment, and their consent was obtained in advance. This study was approved by the Ethics Committee of Chiba University Graduate School of Engineering.

### Stimulus devices

A 21-inch display (resolution 1280 × 1024 pixels, refresh rate 75 Hz) was used for the stimulus presentation. A chin rest was used to fix the participant’s head position, and the viewing distance was 50 cm. Eprime3.0 (Psychology Software Tools, Inc.) was used for all stimulus presentations, including RT reaction time measurements. The participants’ responses were obtained using a numeric keypad.

**Fig. 1:**
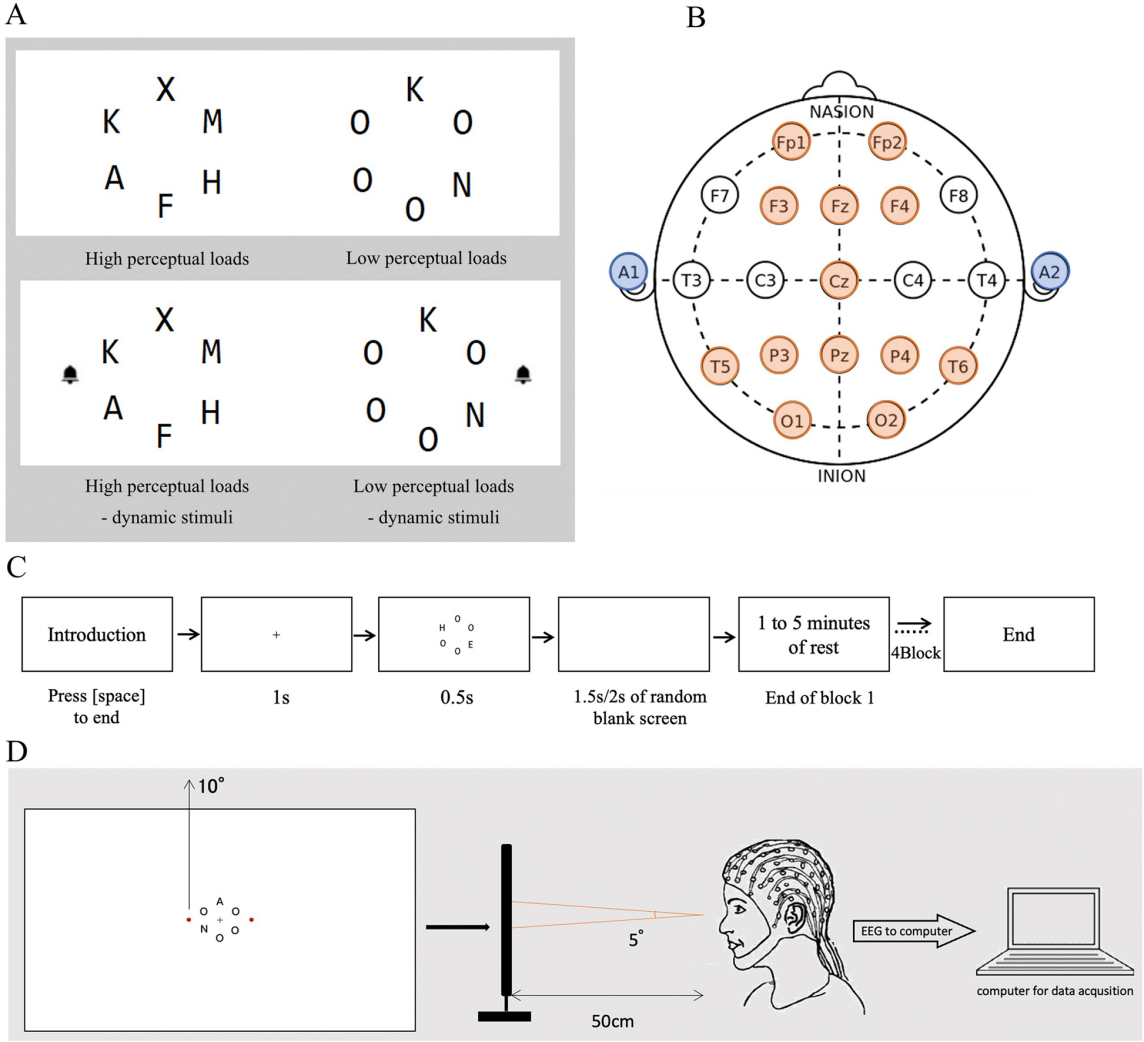
**A** is the type of experimental stimuli with high and low perceptual loads, with and without dynamic stimuli. **B** is the location point of the brainwave acquisition. **C** is the experimental flowchart. **D** is the device’s setup during the experiment, as well as the position of the participants

Stimuli were presented on a white background, The target stimulus was either the letter X or N, while non-target stimuli were randomly selected from A, F, K, H, and M. Each letter subtended approximately 0.87° × 0.94° of visual angle, and a central fixation point (0.7° × 0.7°) was displayed at the center of the screen. In each trial, six letters were arranged in a circular layout with an invisible circle diameter of 4.4 cm, corresponding to a visual angle of 5°. The experiment included 6 conditions: 2 levels of perceptual load (low, high) × 3 dynamic stimulus conditions (none, left visual field, right visual field). Each condition contained 120 target-present trials, with an equal number of target-absent trials, resulting in a total of 1440 trials. Under the low perceptual load condition, four non-target letters were always O, with the remaining two randomly selected from A, K, M, H, or F. Under the high perceptual load condition, all non-target stimuli were selected from A, K, M, H, and F (Fig. 1). The trials were divided into four blocks of 360 trials each, with a 1–5-min rest period between blocks, depending on the participants’ fatigue levels. In this study, dynamic stimulus position (none, left, right) was included as an independent variable, rather than simply distinguishing between “present” and “absent.” This is because visual attention may exhibit spatial asymmetry, meaning that left- and right-side dynamic stimuli could have different effects on attentional allocation and responses. Furthermore, previous studies have suggested that visual processing in the left and right visual fields may be modulated by hemispheric dominance [11]. Therefore, to comprehensively analyze the effects of dynamic stimuli and explore potential interactions, the present study incorporated the “left vs. right” factor into the experimental design. The dynamic stimulus in this experiment was designed to oscillate at 15 Hz, a frequency that falls within the range to which human vision is particularly sensitive [12]. This frequency has been shown effectively captures the attention while also being relatively low, minimizing the risk of visual fatigue and enabling sustained attention over extended periods. A bell icon oscillating at 15 Hz served as the dynamic stimuli and was presented simultaneously with the letter array. It appeared randomly in the left or right visual field at a viewing angle of 10°, functioning as a distractor in the respective condition. Although the stimuli were positioned 10° to the left or right of fixation, they predominantly projected to the contralateral hemisphere and functionally corresponded to the left or right visual field, respectively. Therefore, the terms “left visual field” and “right visual field” are used throughout this manuscript to describe the stimulus locations.

### Experimental procedure

The experimental flow is shown in Fig. 1. At the start of each experiment, the black cross gaze point in the middle of the screen was presented for 1000 ms, followed by the stimulus screen was presented for 500 ms, after which the stimulus screen disappeared, a blank screen was presented for 1500 ms or 2000 ms. Participants were instructed to look for the target stimulus (X, N) while gazing at the black cross gazing point on the stimulus screen and to press the space bar on the keyboard as quickly and accurately as possible. To avoid participants pressing the space bar when they were not observing the target, prior to the experiment, participants performed a practice trial consisting of 20 trials until a correct response rate of 80% was achieved on trials in which the target stimulus appeared. During the practice trials, correct and incorrect feedback was set up, with correct feedback given if the spacebar was pressed when the target stimulus was seen, and incorrect feedback given if the spacebar was pressed when the target stimulus was not seen. In the present experiment, no result feedback was displayed, but false presses were recorded as error trials. RT was calculated as the time from stimulus onset to response execution.

### EEG recording and analysis

**Fig. 2:**
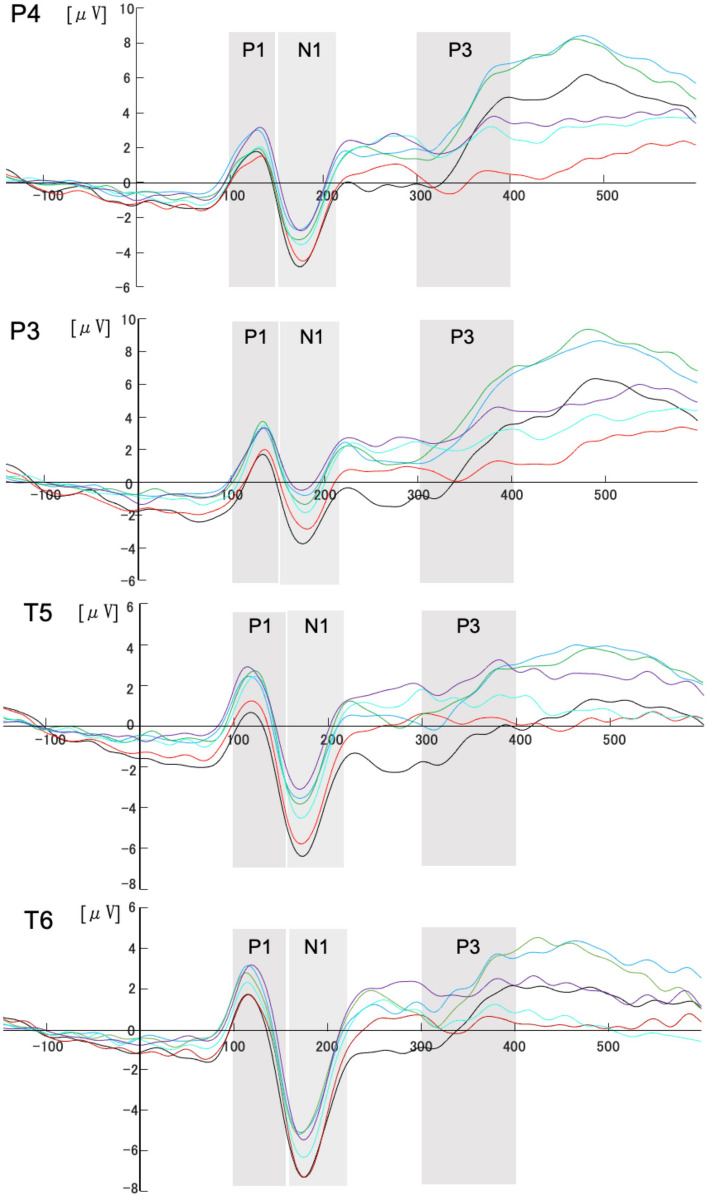
The average amplitude of the P1, N1, and P3 components

Electroencephalography (EEG) was recorded using a BIOPAC system. Measurement sites were Fz, Cz, Pz, Oz, Fp1, Fp2, F3, F4, T5, T6, P3, P4, O1, and O2 sites based on the International 10–20 system (Fig. 1), with averaged ears as reference. Electrooculography (EOG) was recorded to detect blinking with electrodes above and below the right eye. All electrode impedances were below 10 kΩ. EEG signals were recorded at a sampling rate of 1000 Hz and filtered with a low-frequency cutoff of 0.1 Hz and a high-frequency cutoff of 35 Hz. All data analyses were conducted using EEGLAB Toolbox [13] (http://www.sccn.ucsd.edu/eeglab/) and ERPLAB Toolbox, MATLAB-based packages for EEG/ERP data analysis. These signals were low-pass filtered offline using a non-causal Butterworth infinite impulse response filter with a half-power cutoff at 35 Hz and a roll-off of 12 dB/octave. Eye artifact correction was accomplished separately for each participant by subjecting the EEG data recorded to independent components analysis (ICA [14];). Eye artifacts were removed by identifying their topography. After artifact rejection, an average of 78 ± 5 valid trials per condition remained for ERP analysis. The number of valid trials did not differ significantly across conditions. Continuous EEG data were segmented from − 200 ms before stimulus presentation to 600 ms after stimulus onset, with baseline correction applied using the 200 ms period before stimulus presentation (− 200 to 0 ms). Event-related potentials (ERPs) were both referenced to the onset of the circular image containing both target and non-target stimuli, which was set as 0 ms. This study focused on ERP components related to early visual processing (P1, 100–150 ms; N1, 150–220 ms) and attentional resource allocation (P3, 300–400 ms). Since the primary interest was in the magnitude of neural responses under different perceptual load and stimulus conditions, only the amplitude of these components was analyzed. Latency measures were not included, as they were beyond the scope of the current study. Based on the grand averages waveforms and previous research, the waveforms were analyzed separately for P1, N1, and P3 using the Fz, Cz, Pz, Oz, P3, P4, O1, O2, T5, and T6 electrodes. The average amplitude of the P1, N1, and P3 components, as shown in Fig. 2.

### Data analysis

Data were analyzed using repeated-measures analysis of variance (ANOVA) for perceptual load (low, high) and dynamic stimuli (none, left visual field, right visual field) for the experimental conditions. Mauchly’s test of sphericity was employed to assess the assumption of sphericity in the data. In cases where sphericity was found to be violated, adjustments for non-sphericity were performed using the Greenhouse–Geisser, and the corrected p-values were reported with degrees of freedom. The Bonferroni correction was applied to adjust the probability values (p-values) for subsequent analyses [15]. When a significant main effect was observed in the ANOVA, post-hoc analyses were conducted with Bonferroni correction to adjust for multiple comparisons. The significance level was set at α = 0.05. Statistical analyses were performed using SPSS 26.0. All reaction time (RT) and ERP analyses were restricted to correct target trials to ensure that both behavioral and neural measures reflect accurate target processing. RT was calculated from the onset of the circular image containing target and non-target stimuli. Trials with RTs shorter than 100 ms or longer than 800 ms were excluded from analysis (approximately 0.5% of total trials).

## Results

### Behavioral results

For reaction time (RT), the main effect of perceptual load was significant (*F* (1, 13) = 7.850, *p* = 0.017), indicates that participants responded faster under low perceptual load conditions than under high perceptual load conditions. However, the main effect of dynamic stimuli was not significant (*F* (2, 26) = 0.048, *p* = 0.831), nor was the interaction between perceptual load and dynamic stimuli (*F* (2, 26) = 3.047, *p* = 0.109). These results suggest that while high perceptual load significantly slowed response times, the presence of dynamic stimuli did not further modulate this effect, confirming the effectiveness of the perceptual load manipulation in increasing task difficulty (Table 1). For accuracy, neither the main effect of perceptual load nor the main effect of dynamic stimuli was significant (*F* (1, 13) = 2.71, *p* = 0.124, *F* (2, 26) = 2.35, *p* = 0.115, *F*(2, 26) = 0.13, *p* = 0.880). Additionally, the interaction between perceptual load and dynamic stimuli did not reach significance. However, descriptive statistics indicated a trend where accuracy was slightly higher under low perceptual load conditions compared to high perceptual load conditions, although this difference was not statistically significant.

**Table 1:** Mean reaction time (milliseconds) and standard deviation, with percent correct in parentheses, for each trial type

Stimulate	Dynamic stimulation (no)	Dynamic stimulation (left)	Dynamic stimulation (right)
Perceptual load (low)	446.00 ± 5.91 (83%)	441.66 ± 6.55 (84%)	443.42 ± 6.20 (84%)
Perceptual load (high)	446.76 ± 5.13 (79%)	449.86 ± 6.27 (80%)	449.67 ± 5.70 (80%)

### ERP amplitude results

#### P1 statistical analyses

The statistical analysis of the P1 component revealed significant main effects of dynamic stimuli at multiple electrode sites, whereas perceptual load showed no significant main effects. Additionally, no significant interaction between perceptual load and dynamic stimuli was observed at any electrode site (all *p* > 0.1).

Dynamic stimuli elicited a significant main effect at multiple electrode sites, including Fz, P3, P4, O2, T5, and T6 (all *p* < 0.05). Post-hoc analyses indicated that at multiple electrode sites (Fz, P3, T5), P1 amplitude was significantly more positive when dynamic stimuli were presented in either the left or right visual field compared to when no dynamic stimuli were present (all *p* < 0.05). At Cz, P4, O2, and T6, a significantly more positive P1 amplitude was observed only when dynamic stimuli appeared in the right visual field (all *p* < 0.05). Additionally, at O1, a more positive P1 amplitude was found only when dynamic stimuli were presented in the left visual field (*p* = 0.004). Full statistical results are presented in Table 2.

**Table 2:** ANOVA results for P1 component

Electrode	Perceptual load (*F*, *p*)	Dynamic stimuli (*F*,* p*)	Interaction (*F*, *p*)	Post-hoc (dynamic stimuli)
Fz	*F* = 0.645, *p* = 0.436	*F* = 6.775, *p* = 0.004**	*F* = 2.546, *p* = 0.115	Right > None (*p* = 0.004**), Left > None (*p* = 0.029*)
P3	*F* = 0.131, *p* = 0.724	*F* = 8.719, *p* = 0.002**	*F* = 0.394, *p* = 0.672	Right > None (*p* = 0.011*), Left > None (*p* = 0.002**)
P4	*F* = 0.010, *p* = 0.921	*F* = 7.502, *p* = 0.003**	*F* = 0.159, *p* = 0.836	Right > None (*p* = 0.003**)
O2	*F* = 0.021, *p* = 0.888	*F* = 4.737, *p* = 0.026*	*F* = 0.299, *p* = 0.735	Right > None (*p* = 0.003**)
T5	*F* = 1.519, *p* = 0.240	*F* = 10.192, *p* = 0.001**	*F* = 1.192, *p* = 0.315	Right > None (*p* = 0.002**), Left > None (*p* = 0.001**)
T6	*F* = 0.059, *p* = 0.812	*F* = 8.101, *p* = 0.002**	*F* = 0.221, *p* = 0.771	Right > None (*p* < 0.001***)

*F*-values correspond to ANOVA results for perceptual load, dynamic stimuli, and their interaction. Post-hoc comparisons were conducted for dynamic stimuli conditions. Significant results are indicated as follows: *p* <.05*; *p* <.01**; *p* <.001***

#### N1 statistical analyses

The statistical analysis of the N1 component revealed significant main effects of dynamic stimuli at multiple electrode sites, whereas perceptual load showed no significant main effects. Additionally, no significant interaction between perceptual load and dynamic stimuli was observed at any electrode site (all *p* > 0.1).

Dynamic stimuli elicited a significant main effect at multiple electrode sites, including Fz, Cz, Pz, Oz, P3, P4, O1, O2, T5, and T6 (all *p* < 0.05). Post-hoc analyses indicated that at most electrode sites (Fz, Cz, P3, P4, O1, O2, T5, T6), N1 amplitude was significantly more negative when no dynamic stimuli were present compared to when dynamic stimuli appeared in either the left or right visual field (all *p* < 0.05). At Pz and Oz, N1 amplitude was also significantly more negative in the absence of dynamic stimuli compared to the right visual field condition (Pz: *p* = 0.003; Oz: *p* = 0.046). Full statistical results are presented in Table 3.

**Table 3:** ANOVA results for N1 component

Electrode	Perceptual load (*F*, *p*)	Dynamic stimuli (*F*, *p*)	Interaction (*F*, *p*)	Post-hoc (dynamic stimuli)
Fz	*F* = 0.405, *p* = 0.536	*F* = 6.099, *p* = 0.007**	*F* = 2.132, *p* = 0.144	Right < None (*p* = 0.005**), Left < None (*p* = 0.015*)
Cz	*F* = 2.368, *p* = 0.148	*F* = 6.328, *p* = 0.006**	*F* = 0.097, *p* = 0.908	Right < None (*p* = 0.012*), Left < None (*p* = 0.039*)
Pz	*F* = 0.797, *p* = 0.388	*F* = 8.687, *p* = 0.001**	*F* = 0.470, *p* = 0.578	Right < None (*p* = 0.003**)
Oz	*F* = 0.208, *p* = 0.656	*F* = 3.394, *p* = 0.049*	*F* = 0.289, *p* = 0.751	Right < None (*p* = 0.046*)
P3	*F* = 1.912, *p* = 0.190	*F* = 19.688, *p* < 0.001***	*F* = 0.951, *p* = 0.399	Right < None (*p* < 0.001***), Left < None (*p* = 0.005**)
P4	*F* = 0.149, *p* = 0.706	*F* = 13.629, *p* < 0.001***	*F* = 0.067, *p* = 0.873	Right < None (*p* < 0.001***), Left < None (*p* = 0.028*)
O1	*F* = 0.446, *p* = 0.516	*F* = 5.938, *p* = 0.008**	*F* = 0.125, *p* = 0.883	Right < None (*p* = 0.010**), Left < None (*p* = 0.032*)
O2	*F* = 1.569, *p* = 0.232	*F* = 4.660, *p* = 0.020*	*F* = 0.321, *p* = 0.715	Right < None (*p* = 0.016*), Left < None (*p* = 0.041*)
T5	*F* = 0.019, *p* = 0.892	*F* = 21.081, *p* < 0.001***	*F* = 0.949, *p* = 0.393	Right < None (*p* < 0.001***), Left < None (*p* = 0.001**)
T6	*F* = 0.466, *p* = 0.507	*F* = 11.396, *p* < 0.001***	*F* = 0.608, *p* = 0.552	Right < None (*p* < 0.001***), Left < None (*p* = 0.005**)

*F*-values correspond to ANOVA results for perceptual load, dynamic stimuli, and their interaction. Post-hoc comparisons were conducted for dynamic stimuli conditions. Significant results are indicated as follows: *p* <.05*; *p* <.01**; *p* <.001***

Important notice: Since N1 is a negative ERP component, statistical analyses were conducted using negative amplitude values. Importantly, absolute amplitude values were used for post-hoc multiple comparisons to facilitate interpretation

### P3 statistical analyses

The analysis of the P3 amplitude revealed significant main effects of both perceptual load and dynamic stimuli. Perceptual load showed significant effects at multiple sites, including Fz, Cz, Pz, P3, P4, Oz, O1, and O2 (all *p* < 0.05). Post-hoc analysis revealed that at P4, Cz, Pz, Oz, and O2, P3 amplitude in the no dynamic stimulus condition was significantly lower than in the right visual field and left visual field (*p* ≤ 0.003).

Dynamic stimuli elicited a significant main effect at P3, T5, Oz, P4, Fz, Cz, Pz, O1, O2, and T6 (*p* < 0.05), indicating that dynamic stimuli modulated P3 amplitude across widespread scalp regions. Post-hoc analyses indicated that the P3 amplitude was significantly more positive when dynamic stimuli were presented in the right visual field compared to when dynamic stimuli were absent at several electrode sites, including Fz, Cz, Pz, P3, P4, Oz, and T5 (all *p* < 0.05). Moreover, at some electrode sites, including Cz, Oz, P3, P4, and T5, P3 amplitude was significantly more positive when dynamic stimuli were presented in the left visual field compared to when they were absent (all *p* < 0.05). No significant interaction between perceptual load and dynamic stimuli was observed at any electrode site (all *p* > 0.1). A comprehensive summary of the statistical results is provided in Table 4.

**Table 4:** ANOVA results for P3 component

Electrode	Perceptual load (*F*, *p*)	Dynamic stimuli (*F*, *p*)	Interaction (*F*, *p*)	Post-hoc (dynamic stimuli)	Post-hoc (perceptual load)
Fz	*F* = 14.069, *p* = 0.002**	*F* = 5.599, *p* = 0.010*	*F* = 0.708, *p* = 0.502	Right > None (*p* = 0.006**)	Left > None (*p* = 0.001**)
Cz	*F* = 18.881, *p* = 0.001**	*F* = 5.100, *p* = 0.014*	*F* = 0.966, *p* = 0.394	Right > None (*p* = 0.02*), Left > None (*p* = 0.05*)	Right > None (*p* = 0.003**), Left > None (*p* = 0.001**)
Pz	*F* = 16.322, *p* = 0.001**	*F* = 4.359, *p* = 0.023*	*F* = 1.260, *p* = 0.295	Right > None (*p* = 0.017*)	Right > None (*p* = 0.016*), Left > None (*p* = 0.001**)
Oz	*F* = 11.786, *p* = 0.004**	*F* = 6.752, *p* = 0.004**	*F* = 1.189, *p* = 0.321	Right > None (*p* = 0.002**), Left > None (*p* = 0.05*)	Left > None (*p* = 0.001**)
P3	*F* = 2.826, *p* = 0.117	*F* = 11.891, *p* < 0.001***	*F* = 2.253, *p* = 0.125	Right > None (*p* < 0.001***), Left > None (*p* = 0.004**)	
P4	*F* = 40.942, *p* < 0.001***	*F* = 7.590, *p* = 0.003**	*F* = 0.760, *p* = 0.478	Right > None (*p* = 0.002**), Left > None (*p* = 0.038*)	Right > None (*p* < 0.001***), Left > None (*p* < 0.001***)
O1	*F* = 5.163, *p* = 0.041*	*F* = 5.028, *p* = 0.014*	*F* = 0.976, *p* = 0.390	Right > None (*p* = 0.023*), Left > None (*p* = 0.017*)	Left > None (*p* = 0.03*)
O2	*F* = 10.850, *p* = 0.006**	*F* = 4.220, *p* = 0.026*	*F* = 0.416, *p* = 0.664	Right > None (*p* = 0.008**)	Right > None (*p* = 0.029*), Left > None (*p* = 0.002**)
T5	*F* = 0.966, *p* = 0.344	*F* = 8.754, *p* = 0.001**	*F* = 0.694, *p* = 0.509	Right > None (*p* = 0.003**), Left > None (*p* = 0.01*)	
T6	*F* = 2.522, *p* = 0.136	*F* = 5.358, *p* = 0.011*	*F* = 0.625, *p* = 0.543	Right > None (*p* = 0.001**)	

*F*-values correspond to ANOVA results for perceptual load, dynamic stimuli, and their interaction. Post-hoc comparisons were conducted for dynamic stimuli conditions. Significant results are indicated as follows: *p* <.05*; *p* <.01**; *p* <.001***

## Discussion

This exploratory study examined the behavioral and neurophysiological effects of dynamic stimuli on visual attentional selection under varying perceptual loads using ERP recordings. Task performance showed that high perceptual load prolonged reaction times during visual search, whereas a trend suggestive of attentional capture reversal emerged under low perceptual load. ERP results showed that dynamic stimuli enhanced early sensory processing, as evidenced by increased P1 amplitude, while the N1 amplitude was more negative when dynamic stimuli were absent. In contrast, the P3 component appeared to reflect perceptual load-dependent integration, with notably greater positivity observed under low load and in the presence of dynamic stimuli, consistent with previous findings that P3 amplitude is sensitive to task demands and mental workload during post-perceptual processing [9, 16].

Task performance, indexed by reaction time, systematically varied with load levels. Higher perceptual load prolonged reaction times in trials with dynamic stimuli, consistent with prior findings on resource depletion under high load [10]. However, the interaction between perceptual load and dynamic stimuli was not statistically significant in the present study. Nevertheless, under low perceptual load, reaction times were paradoxically shorter in trials with dynamic stimuli than in those without—a phenomenon known as “attentional capture reversal” [17]. Although previous studies, such as [10], reported significant interactions between perceptual load and distractor interference, the interaction was not observed here. This discrepancy may be attributed to the high salience and spatial distinctiveness of dynamic distractors used in the present study. Additionally, differences in task demands and participants’ strategic attentional control might have reduced the observable interaction effect. This reversal suggests that salient distractors facilitate search efficiency when residual resources enable proactive suppression of task-irrelevant signals. The modest (~ 10 ms) reduction in search time with distractors aligns with research showing that under low load, salient distractors can be actively suppressed, reducing interference [18], likely reflecting strategic reallocation of attention: participants prioritized target features (e.g., “X”/“N”) while inhibiting spatially distinct dynamic distractors, thereby reducing interference. This pattern aligns with the Guided Search Theory [19], which posits that distractors are suppressed more effectively when perceptual load is low. Recent studies further demonstrate that distractor presence can paradoxically enhance performance when suppression mechanisms are effectively deployed [8, 20, 21]. In this task, the perceptual salience of dynamic distractors (compared to static backgrounds) likely allowed participants to rapidly exclude task-irrelevant regions, narrowing attentional focus to target locations.

The ERP results revealed robust effects of dynamic stimuli on early sensory and attentional components. P1 amplitude was significantly enhanced in the presence of dynamic stimuli, reflecting the heightened salience of motion stimuli in early visual processing [22, 23]. This enhancement aligns with theories of sensory gain control model, which suggests that visually salient transients automatically amplify early-stage sensory processing, even when they are task-irrelevant stimuli [24].

Conversely, N1 amplitude was more negative in trials without dynamic stimuli, suggesting suppression of the fixed distractor location (10° visual angle) when no salient motion cues were available to guide attention [25]. Two complementary mechanisms may account for this effect. First, the consistent spatial location of distractors under without dynamic stimuli conditions likely increased spatial predictability, promoting anticipatory attentional allocation and enhancing early sensory responses [24, 26]. Second, the absence of dynamic elements made the visual display more uniform, reducing competition from background features and improving the distinction between target and distractor, thereby enhancing the N1 response [27]. Together, these factors likely enabled participants to more effectively suppress interference from the predictable distractor area, resulting in greater N1 amplitudes in visually stable contexts [28].

The dissociable effects on P1 and N1 components underscore their distinct functional roles. The enhanced P1 amplitude observed in response to dynamic stimuli likely reflects sensitivity to motion-related salience, which aligns with the magnocellular pathway’s preferential processing of transient visual signals [22]. In contrast, more negative N1 amplitude in trials without dynamic stimuli may reflect efforts to limit interference from the consistently positioned distractors [25]. This pattern is consistent with hierarchical models of attentional selection, in which bottom-up attentional capture (reflected by P1) occurs first, followed by goal-directed attentional suppression (indexed by N1) [2]. Such a two-stage mechanism aligns with findings that early sensory processing is prioritized for visually salient stimuli, whereas later processing depends on task demands and suppression strategies [20].

The absence of perceptual load effects on P1 and N1 components contrasts with traditional load theory [29] but aligns with studies showing that intermixed trial designs attenuate load-dependent modulation [30]. Random presentation of high/low load trials likely prompted participants to adopt a default attentional window, minimizing sensitivity to load variations [31]. Furthermore, dynamic distractors may override top-down load effects, as motion cues engage automatic attentional capture regardless of resource availability [20]. This suggests dynamic stimuli dominate early attentional selection, while perceptual load operates later in processing (e.g., P3-stage resource integration).

The P3 component, a hallmark of late-stage attentional resource allocation, exhibited significant sensitivity to both perceptual load and dynamic stimuli. Under high perceptual load, P3 amplitude was markedly reduced, likely because increased task demands depleted cognitive resources, leaving fewer available for target identification. This is consistent with prior findings that P300 amplitude inversely correlates with perceptual load, reflecting a “resource depletion” mechanism [32, 33].

Conversely, the presence of dynamic stimuli significantly enhanced P3 amplitude across both high and low load conditions. This suggests that motion salience enables attentional capture even when resources are constrained. Dynamic stimuli may function as attention-grabbing signals, diverting resources toward their processing despite competing task demands. The paradoxical increase in P3 amplitude under high load suggests that motion salience can override top-down resource limitations, potentially compelling the cognitive system to either integrate or suppress these salient distractors [9, 20].

These findings suggest that the P3 component may be important for regulating attentional resource allocation. While high perceptual load reduces available attentional resources (lowering P3 amplitude), dynamic stimuli seem to locally capture attention, leading to an increase in P3 amplitude. This tension aligns with theories positing that P3 amplitude reflects the balance between task-relevant resource investment and distractor-driven interference [9]. However, the precise relationship between the P3 component and the perceptual load remains debated. Some studies question whether P3 amplitude is a reliable indicator of perceptual load [34–36], suggesting that it primarily reflects stimulus evaluation rather than attentional capacity. Conversely, other studies, including this one, demonstrate that the P3 component can be sensitive to load effects, particularly when combined with salient distractors [32, 37]. This suggests the P3 component may not simply reflect overall resource depletion but rather the dynamic interplay between task demands and stimulus-driven attentional capture. These findings provide insights into how humans dynamically allocate attentional resources to adapt to complex environmental demands. ERP results further illustrate the impact of dynamic stimuli on neural processing. The modulation of P1 and N1 components suggests that perceptual load influences visual attention not by altering early sensory responses but by regulating the balance between stimulus-driven salience and task-related suppression processes. This highlights the role of dynamic stimuli in attentional adaptation and contributes to a deeper understanding of the physiological basis of cognitive flexibility in response to changing environmental demands.

### Limitations

This study has several limitations. First, the mixed-block design may have led to uniform attention, reducing differences in early ERP components across perceptual load conditions. The study focused solely on visual stimuli, excluding multimodal inputs like auditory or tactile cues. Future research could explore multimodal effects. Furthermore, ERP was used as the sole physiological measure, which may have overlooked other neural or behavioral processes associated with attention modulation. Incorporating additional methods, such as eye-tracking or functional MRI, could provide richer insights into the mechanisms of attention.

## Conclusion

This study employed the perceptual load paradigm to investigate the effects of dynamic stimuli on attention under different perceptual load conditions, analyzing task performance and ERP components. Task performance showed that high perceptual load prolonged reaction times during visual search, whereas a trend suggestive of attentional capture reversal emerged under low perceptual load. ERP findings indicated that P1 and N1 were insensitive to perceptual load but were influenced by the presence of dynamic stimuli. Specifically, P1 amplitude increased when dynamic stimuli were present, whereas N1 amplitude was more negative in their absence. P3 amplitude decreased under high perceptual load, indicating the need to suppress interference from dynamic stimuli during target selection. Given their salient visual properties, dynamic stimuli can trigger bottom-up attentional capture and distraction, thereby exacerbating visual attention interference and complicating visual processing.

## Data Availability

No datasets were generated or analysed during the current study.

## Abbreviations

ANOVA: Analysis of Variance
ERP: Event-related potential
EEG: Electroencephalography
RT: Reaction times
